# Comparative functional and evolutionary analysis of essential germline stem cell genes across the genus *Drosophila* and two outgroup species

**DOI:** 10.1093/g3journal/jkaf224

**Published:** 2025-10-03

**Authors:** Luke R Arnce, Jaclyn E Bubnell, Charles F Aquadro

**Affiliations:** Department of Molecular Biology and Genetics, Cornell University, Ithaca, NY 14853, United States; Department of Molecular Biology and Genetics, Cornell University, Ithaca, NY 14853, United States; Department of Molecular Biology and Genetics, Cornell University, Ithaca, NY 14853, United States

**Keywords:** bam, germline stem cells, comparative functional analysis, CRISPR

## Abstract

In *Drosophila melanogaster*, *bag of marbles* (*bam*) encodes a protein essential for germline stem cell (GSC) daughter differentiation in early gametogenesis. Despite its essential role in *D. melanogaster*, direct functional evaluation of *bam* in other closely related *Drosophila* species reveal this essential function is not necessarily conserved. In *Drosophila teissieri*, for example, *bam* is not essential for GSC daughter differentiation. Here, we generated *bam* null alleles using CRISPR-Cas9 in a species more distantly related to *D. melanogaster*, *Drosophila americana*, to interrogate whether *bam*'s essential GSC differentiation function is novel to the *melanogaster* species group or a function more ancestral to the *Drosophila* genus. To further characterize the extent of the functional flexibility of other GSC-regulating genes, we generated a gene ortholog dataset for 366 GSC-regulating genes essential in *D. melanogaster* across 15 additional *Drosophila* and two outgroup species. We find that *bam*'s essential GSC function is conserved between *D. melanogaster* and *D. americana* and therefore originated prior to the formation of the *melanogaster* species group. Additionally, we find that ∼8% of the 366 GSC genes essential in *D. melanogaster* are absent in at least one of the 17 species in our ortholog dataset. These results indicate that developmental systems drift, in which the specific genes regulating a function may change, but the final phenotype is retained, occurs in stem cell regulation and the production of gametes across *Drosophila* species.

## Introduction

Proper production of gametes is critical for reproduction, and in *Drosophila* it begins with the asymmetric division of germline stem cells (GSCs) to both self-renew, maintaining the germline, and differentiate to produce sperm and eggs (Kahney et al. 2019). Misregulation of this highly sensitive process can quickly lead to sterility, so coordination of these early cellular divisions might be presumed to be highly conserved (Gleason et al. 2018). However, recent results have demonstrated that several GSC-regulating genes essential for reproduction in *Drosophila melanogaster* show signs of positive selection through rapid amino acid diversification, are nonessential for fertility, and/or are completely absent in non- *melanogaster Drosophila* species (Civetta et al. 2006; Bauer DuMont et al. 2007; Choi and Aquadro 2015; Flores et al. 2015; DuMont et al. 2021; Bubnell et al. 2022).

One of these GSC-regulating genes is bag-of-marbles (*bam*). In *D. melanogaster*, the encoded protein Bam is 442 amino acids with several known functions that are executed in complexes with other protein partners. The most well characterized of Bam's functions is as the switch for GSC daughter differentiation, but Bam also has documented roles in the maintenance of gut integrity and as a switch for preventing premature differentiation of hematopoietic progenitor cells (McKearin and Spradling 1990; Insco et al. 2009; Tokusumi et al. 2011).

*Bam* specifically acts as the switch gene for GSC differentiation for *D. melanogaster* females and is necessary for terminal differentiation of spermatogonia in males (McKearin and Spradling 1990). In females, *bam* is repressed in GSCs, and bam expression causes differentiation by binding to several protein partners including *benign gonial cell neoplasm* (Bgcn) to repress the production of self-renewal factors Nanos and elF4a. The resulting differentiating cystoblast undergoes several mitotic divisions (Ohlstein et al. 2000; Li et al. 2009; Shen et al. 2009; Li et al. 2013). Simultaneously, Bam concentrates at the fusome, which connects the developing cystoblasts, and Bam and Bgcn function together to regulate the timing of mitotic divisions between cells. In males, Bam is expressed in GSCs, and as differentiation continues, expression increases (McKearin and Spradling 1990; Pan et al. 2014; Ji et al. 2017; Sgromo et al. 2018). Once *bam* expression reaches a threshold in the early spermatogonia, Bam binds to Bgcn and tumorous testis (*tut*) (Insco et al. 2009; Insco et al. 2012; Ting 2013). Binding represses *mei-P26* and ends proliferation, triggering terminal differentiation and beginning meiosis (Ohlstein et al. 2000; Li et al. 2009; Shen et al. 2009; Li et al. 2013). Loss of *bam* function prevents differentiation in both sexes and causes overproliferation of GSCs in females and spermatogonia in males, leading to tumors and sterility in *D. melanogaster* (Ohlstein and Mckearin 1997; Shivdasani and Ingham 2003).

**Fig. 1.**
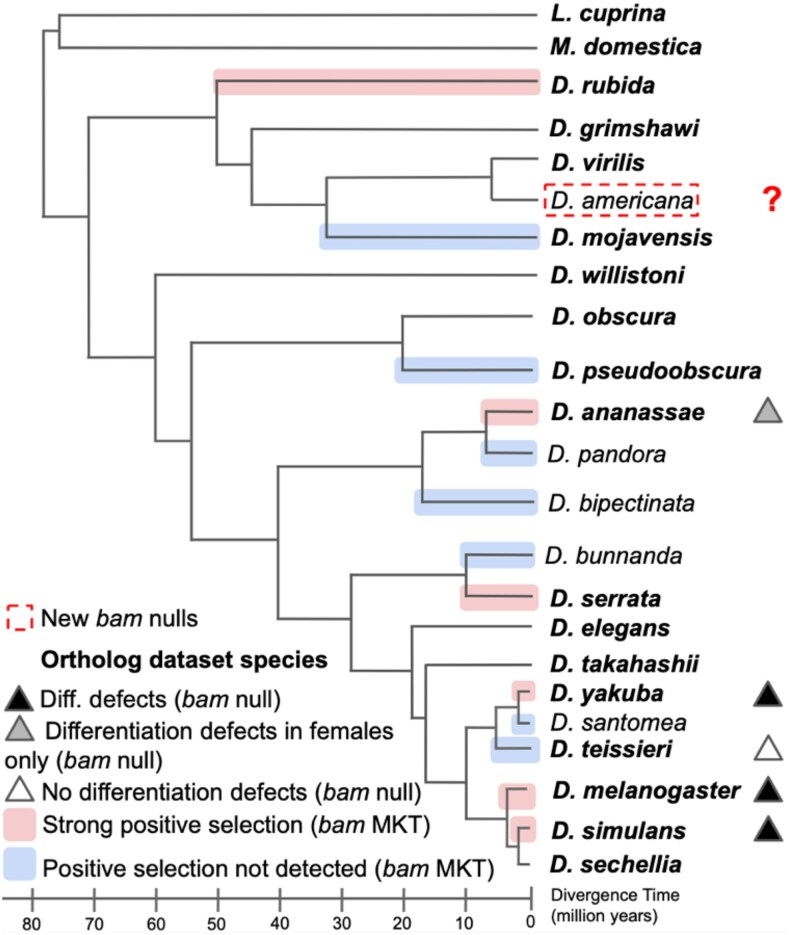
Phylogeny of select *Drosophila* and outgroup species with relevant functional and evolutionary data for *bam* partially adapted from Bubnell et al. (2022). The scale below the phylogeny indicates the time since divergence from *D. melanogaster* (MY) inferred by Li et al. (2021) from sequence divergence and the rate of genomic mutation molecular clock estimated by Tamura et al. (2004). Divergence times from *D. melanogaster* for *Musca* and *Lucilia* were estimated by Weigmann et al. (2003). Note that there are other estimates of divergence times in the literature that give different absolute dates and that depend on other estimated rates or calibrations (e.g. Tamura et al. 2004; Markow and O’Grady 2006; Obbard et al. 2012; Suvorov et al. 2022).

Though *bam* plays an essential role in GSC regulation in *D. melanogaster*, *bam*'s sequence and function vary considerably across *Drosophila* or outgroup species (Bubnell et al. 2022). The total 442 amino acid Bam protein in *D. melanogaster* differs by 60 fixed amino acid differences (∼14%) with its sibling species *Drosophila simulans*. Bam sequences differing from *D. melanogaster* by up to 308 (67%) of amino acids in other *Drosophila* species and up to 87% in outgroup species *Musca domestica* and *Lucilia cuprina* (Arnce et al. 2025). Statistical tests of selection for *D. melanogaster* and *D. simulans bam* suggest that 94% and 72% of fixed amino acid differences respectively were driven by natural selection between these two species and their common ancestor (Bubnell et al. 2022). Additional signals of positive selection at *bam* were detected in other *Drosophila* lineages across the genus leading to *Drosophila yakuba*, *Drosophila ananassae*, and *Drosophila rubida*, for example, while other lineages, despite evaluation, showed no evidence of positive selection for protein diversification (Fig. 1.) (Bubnell et al. 2022). These data suggest a striking level of sequence divergence, much of which was potentially driven by natural selection, in orthologs of a gene critical for ensuring fertility in a *D. melanogaster*.

Additionally, recent functional studies of *bam* using complete loss-of-function (null) alleles in *Drosophila* species (Bubnell et al. 2022) (Fig. 1.) revealed divergent roles across *D. simulans*, *D. melanogaster*, *Drosophila teissieri*, *D. yakuba*, and *D. ananassae*. In *D. teissieri bam* null mutants showed no GSC differentiation defects in either sex, suggesting *bam* lacks its canonical role as a critical differentiation regulator in this species. While in *D. ananassae bam* null females were sterile, males exhibited normal spermatogenesis. These results demonstrate evolutionary divergence in *bam* sequence and function, despite its conserved essential role in GSC regulation in other *Drosophila* lineages.

Analyses of DNA polymorphism and divergence among other GSC regulatory genes within the *D. melanogaster* species group have revealed distinct signatures of adaptive evolution across lineages (Bubnell et al. 2022) (Fig. 1). Notably, some core components of GSC regulatory networks exhibit dynamic evolutionary trajectories, including lineage-specific gene loss. For example, *Yb*—a gene expressed in somatic cap cells of the germarium and essential for GSC maintenance and transposable element suppression in *D. melanogaster* (Szakmary et al. 2009)—shows pronounced amino acid divergence between *D. melanogaster* and *D. simulans* (Flores et al. 2015). While *Yb* null mutants in *D. melanogaster* result in female sterility (Swan et al. 2001); Chary and Hayash (2023) have recently reported that *Yb* is absent from the genomes of *Drosophila eugracilis*, *Drosophila pseudoobscura*, and *Drosophila persimilis*. These findings underscore both the evolutionary plasticity of GSC regulation and the potential for critical gene turnover within conserved developmental pathways.

A persistent challenge in comparative functional genetics lies in the widespread assumption of ortholog functional conservation, despite limited empirical validation. Most studies focus on single-species models, leaving ortholog activity across taxa largely inferred rather than experimentally confirmed (Tekaia 2016). Systematic assessment of the mechanistic basis and degree of functional conservation is crucial for reconstructing the evolution of gene networks. This limited validation of ortholog conservation introduces significant limitations when interpreting comparative genomic data: nonconserved ortholog functions imply divergent evolutionary pressures across lineages, thereby confounding hypotheses about ancestral genetic architectures or drivers of molecular evolution. Recent studies in bacteria and *Diptera* have begun to evaluate conservation of functional genes across closely related species and have identified surprising variability in ortholog functional conservation (Bergmiller et al. 2012; Carranza et al. 2018; Leeuwen et al. 2020; Deng et al. 2024; Hopkins et al. 2024; Zakerzade et al. 2025). Lineage-specific ortholog loss of functional reproductive genes has also been observed between humans and nonhuman primates (Carlisle et al. 2024). Altogether, these results suggest a more comprehensive comparative functional analysis of *bam*, and other essential GSC-regulating genes across the genus *Drosophila* could provide insight into the functional evolutionary history and extent of network flexibility of bam and essential GSC genes more broadly.

We executed our comparative functional analysis of *D. melanogaster*-essential GSC-regulating genes using a two-pronged approach: generating a bam null allele in an additional divergent *Drosophila* species and evaluating ortholog presence or absence across 15 diverse *Drosophila* and two outgroup species (sheep blowfly *L. cuprina* and house fly *M. domestica*) (Fig. 1).

We chose to generate a *bam* null mutant *in Drosophila americana* as this species represents a major, more divergent outgroup lineage to the *D. melanogaster* species group within the *Drosophila* genus (*D. americana and D. melanogaster* diverged approximately 70 MYA) (Fig. 1) and has previously been successfully edited with CRISPR/Cas9 (Vankuren and Long 2018; Lamb et al. 2020). We evaluated cytology and fertility in this null mutant using the strategy from Bubnell et al. (2022) to evaluate *bam*'s function in GSC differentiation. This analysis adds broader evolutionary scope to our knowledge of *bam* function and provides additional insight into whether *bam*'s essential role in GSC differentiation is likely ancestral to all *Drosophila* species and was lost in specific lineages or whether *bam*'s critical role was a gained function within the *D. melanogaster* species group. Defining *bam* null phenotypes also provide a broader context for understanding the relationship between *bam* function and positive selection.

Null mutants are effective for performing comparative analyses of function for genes, like *bam*, with orthologs across species of interest. However, generating null mutants is expensive and time consuming in nonmodel species. Identifying ortholog absences in other species for GSC-regulating genes essential in *D. melanogaster* provides an alternative rapid and cost-effective strategy for identifying potential functional differences in the genes and interaction networks that regulate GSC maintenance, self-renewal, and differentiation. To investigate ortholog functional conservation via ortholog presence or absence, we started with a set of 366 GSC-regulating genes determined to be essential in *D. melanogaster* from a functional RNAi screen (Yan et al. 2014) along with two of *bam*'s close interacting partners that are also essential for fertility in *D. melanogaster*: *bgcn* and *Yb* (Li et al. 2009; Szakmary et al. 2009). We then used orthology tools in conjunction with a custom reciprocal best blast hit (RBBH) pipeline, a common ortholog identification strategy (Moreno-Hagelsieb et al. 2008; Kristensen et al. 2011; Wolf et al. 2012), to identify ortholog presences/absences across 15 diverse *Drosophila* and two outgroup species. We also used the custom pipeline to identify physical and genetic GSC interacting gene orthologs and validate ortholog absences using a combination of localized PCR and sequencing when possible. Finally, we integrated functional categories as identified in Yan et al. (2014) for the 366 included GSC-regulating genes. This generated gene ortholog dataset enables evaluation of the extent and characteristics of essential GSC-regulating gene network flexibility as well as informed predictions regarding their functional evolutionary histories.

Here, we report that both female and male *D. americana bam* null mutants are sterile with GSC regulation defects, indicating *bam*'s essential GSC-regulating function is not novel to the *melanogaster* species group, is likely ancestral to the genus Drosophila, and provides additional evidence that species in which *bam* is not necessary for gametogenesis (e.g. *D. teissieri* and *D. ananassae*) represent lineage-specific functional losses. Our comparative ortholog analysis of GSC-regulating genes reveals that there is additional functional flexibility beyond *bam* with ∼8% (30 of 366) of the genes absent in one or more of the 17 included species and ∼3% absent in one or more of the 15 included *Drosophila* species. We also confirm that *Yb* is missing from *D. pseudoobscura* and find it missing in the *Drosophila obscura* genome, despite it being necessary for GSC function and development, and therefore fertility, in *D. melanogaster*. Ortholog conservation does not necessarily indicate conservation of function (e.g. *bam*), so this represents the minimum functional flexibility in essential GSC-regulating genes. These results altogether are consistent with other recent studies showing genes essential in one species for critical functions, like fertility, do not necessarily have the same essential function even among closely related species (Leeuwen et al. 2020). *Bam* functional variation and the absences of several essential GSC-regulating gene across *Drosophila* are potential examples of developmental systems drift (DSD) or divergence in genetic systems that underpin a conserved phenotype (Weiss and Fullerton 2000; True and Haag 2001).

## Materials and methods

### Fly stocks and rearing

We raised fly stocks on standard cornmeal-molasses food at room temperature, and we used yeast-glucose food for fertility assays. We acquired lines with sequenced genomes for 15 *Drosophila* and two outgroup species: *D. simulans* (strain: w501), *D. sechellia* (strain: sech25), *D. teissieri* (strain: GT53w), *D. yakuba* (strain: Tai18E2), *Drosophila takahashii* (strain: IR98-3 E-12201), *Drosophila elegans* (strain: 14027-0461.03), *Drosophila serrata* (strain: Fors4), *D. ananassae* (strain: 14024-0371.14), *D. pseudoobscura* (strain: MV2-25), *D. obscura* (strain: BZ-5 IFL), *Drosophila willistoni* (strain: 14030-0811.24), *Drosophila mojavensis* (strain: 15081-1352.22), *Drosophila virilis* (strain: 15010-1051.87), *Drosophila grimshawi* (strain: 15287-2541.00), *D. rubida* (strain: PH 161), *M. domestica* (strain: aabys), and *L. cuprina* (strain: Lc7/37) (Supplementary File 1 Table A). We also acquired *D. simulans*, *sechellia*, *yakuba*, *serrata*, *willistoni*, *mojavensis*, *virilis*, and *grimshawi* from the National *Drosophila* Species Stock Center (NDSSC) (http://blogs.cornell.edu/drosophila/), *D. takahashii* from Kyorin-fly *Drosophila* species stock center (https://shigen.nig.ac.jp/fly/kyorin/), and *D. teissieri*, *D. pseudoobscura*, the house fly *M. domestica*, and the sheep blowfly *L. cuprina* as gifts from Daniel Matute, Andrew G. Clark, Jeffrey Scott, and Max Scott, respectively. *D. elegans* and *D. ananassae* were gifts from Artyom Kopp. *D. obscura* and *D. rubida* were gifts from Dmitri Petrov. We acquired the *D. americana* white eye mutant line (Lamb et al. 2020) for null generation as a gift from Trisha Wittkopp (Supplementary File 1 Table A).

### Strategy for generating a *bam* null phenotype in *D. americana*

We generated the *bam* null disruption by targeting the first exon of *bam* and introducing an early stop codon in the coding sequence using CRISPR/Cas9 gene editing. Because null homozygotes are sterile and thus cannot be maintained, we developed two *bam* disruption lines (one marked by 3 × 3P-Dsred and the other by 3xP3-YFP) which can be maintained as heterozygous lines (Supplementary File 1 Tables B–F). In order to phenotype the null homozygote, we crossed *bam*^3xP3−Dsred^/*bam^wt^* and *bam*^3xP3−YFP^/*bam^wt^* flies to create a *bam* disruption null homozygotes (*bam*^3xP3−Dsred^/*bam^YFP^*) which we then identified via fluorescent eye screen.

### Bam null construct cloning

Yasir Ahmed gifted us *bam* nucleotide sequences for *D. americana* (now on NCBI as G96 accession: PRJNA475270), and we performed cloning design in Geneious (Supplementary File 1 Table D). We generated PCR products using the NEB Q5 High Fidelity 2x master mix, then gel extracted and purified using the NEB Monarch DNA gel extraction kit. For PCR, sequencing, and cloning, we used IDT primers. We also generated donor plasmids for both the 3xP3-YFP and 3xP3-Dsred *bam* disruption lines using the strategy outlined in (Bubnell et al. 2022). We prepared and purified plasmids for embryo injections with the Qiagen plasmid plus midi-prep kit followed by phenol-chloroform extraction for further RNase removal and then sequenced plasmids with whole plasmid sequencing (Plasmidsaurus).

### CRISPR/Cas9 and gRNA selection

We used Geneious to select gRNAs with no predicted off-targets in the reference genomes for *D. americana* (Supplementary File 3). We generated these as synthetic gRNAs (sgRNAs) from Synthego and used up to two gRNAs per injection to improve the likelihood of successful CRISPR events (Supplementary File 1 Table F).

### Embryo injections

Genetivision performed CRISPR/Cas9 injections including the appropriate plasmid donor, sgRNAs, and Cas9 protein (Synthego) into the *D. americana* line. We screened *bam* disruption lines for eye color to identify CRISPR/Cas9 mutant flies in-house using a Nightsea fluorescence system with YFP (cyan) and DsRed (green) filters. We backcrossed positive flies to generate lines which were maintained as heterozygous stocks. We confirmed CRISPR insertions by sequencing (Plasmidsaurus).

### Fertility assays

We executed following the strategy from Flores et al. (2015) for all female fertility assays. We collected and aged virgin females to sexual maturity (3–4 d for *D. americana*) (Markow and O’Grady 2006). We collected all generated genotypes from each bottle to control for bottle effects. Wild-type virgin males for each species were also aged until sexual maturity and distributed from different bottles across female genotypes. We crossed single females with two males, allowed to mate for nine days, then flipped onto new vials for nine more days, and then finally cleared from the vials while the offspring develop. We counted progeny daily and cleared to get total adult progeny per female. We also conducted male fertility assays with wild-type females and males of all generated genotypes. We executed fertility assays for *D. americana* on yeast-glucose food and all fertility experiments were kept at room temperature (approximately 21 °C).

### Fertility assay statistics

We used estimation statistics to assess fertility assay mean difference (effect size) in the number of adult progeny between the wild-type *bam* genotype and the *bam* null homozygote and heterozygote genotypes. We generated estimation statistics and shared control Cumming plots using www.estimationstats.com (Ho et al. 2019) (github.com/lukearnce/bam_null_ortholog). We used estimation statistics to enable determination of the size of the impact of the *bam* genotype on fertility via a non-parametric methodology. We reported significance as an effect size outside the 95% confidence interval (github.com/lukearnce/bam_null_ortholog).

### Immunostaining

We used the following primary antibodies: anti-Hts-1B1 (mouse, AB_528070, Developmental Studies Hybridoma Bank, concentrate 1:40) and anti-vasa (rat, AB_760351, DSHB, concentrate 1:20. We also used the following secondary antibodies: Alexaflour goat anti-rat 488 and goat anti-mouse 568 (Invitrogen) at 1:500. We performed immunostaining as described in Bubnell et al. (2022). In short, we dissected ovaries and testes in cold 1× PBS and pipetted up and down to improve antibody permeability, fixed tissues in 4% paraformaldehyde, washed in PBST (1× PBS, 0.2% Triton-X 100), blocked in PBTA (1× PBS, 0.2% Triton-X 100, 3% BSA) (Alfa Aesar), and, next, incubated in the appropriate primary antibody in PBTA overnight. We washed (PBST), blocked (PBTA), and incubated tissues in the appropriate secondary antibody for two hours. The tissue was then washed again (PBST) and finally mounted in mounting media with DAPI (Prolong glass antifade with NucBlue, Invitrogen) for imaging.

### Microscopy

We imaged ovaries and testes on a Zeiss i880 confocal microscope with 405, 488, and 568 nm laser lines at 40× (Plan-Apochromat 1.4 NA, oil) (Cornell BRC Imaging Core Facility). We analyzed and edited images using Fiji (ImageJ).

### McDonald–Kreitman test (MKT) of selective neutrality for *bam* in the *D. americana* lineage

We tested *D. americana bam* for significant departures from neutrality via the MKT (McDonald and Kreitman 1991). *D. americana bam* polymorphism and the *D. lummei*, and *D. virilis* sequences used to assess divergence were gifted by Yasir Ahmed. We implemented the strategy for MKT analysis of *D. americana bam* from Bubnell et al. (2022). In brief, we aligned *bam* sequences using PRANK (webPRANK accessed 07-14-25) (Löytynoja and Goldman 2010) with the -codon and -F parameters using the PRANK tree guide. We used the codeml package from PAML (version 4.9) (Yang et al. 2007) to generate the predicted common ancestor sequences for calculating lineage-specific divergence for *bam* with the MKT. We used PRANK alignments and trees as inputs to codeml with control file parameters (noisy = 9, verbose = 2, runmode = 0, seqtype = 1, CodonFreq = 2, clock = 0, aaDist = 0, model = 0, NSsites = 0, icode = 0, getSE = 0, RateAncestor = 1, Small_diff = 0.5e-6, cleandata = 0, method = 1) (github.com/lukearnce/bam_null_ortholog). We conducted an MKT comparing nonsynonymous and synonymous changes (Egea et al. 2008). We excluded polymorphic sites at less than 12% frequency, classified as slightly deleterious alleles not yet removed by purifying selection (Charlesworth and Eyre-Walker 2008). We used the predicted common ancestral *bam* sequence of *D. americana* and *D. lummei* (with *D. virilis* as the outgroup) to calculate lineage-specific divergence. We recorded values for the contingency table and the *P*-value of the two-tailed Fisher's exact test (github.com/lukearnce/bam_null_ortholog).

### Genomes used in analysis

We downloaded genomes from NCBI for 15 *Drosophila* and two outgroup species: *D. simulans* (assembly: Prin_Dsim_3.1), *D. sechellia* (assembly: ASM438219v2), *D. teissieri* (assembly: Prin_Dtei_1.1), *D. yakuba* (assembly: Prin_Dyak_Tai18E2_2.1), *D. takahashii* (assembly: ASM1815269), *D. elegans* (assembly: ASM1815250), *D. serrata* (assembly: Dser1.1), *D. ananassae* (assembly: ASM1763931v2)*, D. pseudoobscura* (assembly: UCI_Dpse_MV25), *D. obscura* (assembly: ASM1815110v1), *D. willistoni* (assembly: UCI_dwil_1.1), *D. mojavensis* (assembly: ASM1815372v1), *D. virilis* (assembly: Dvir_AGI_RSII-ME), *D. grimshawi* (assembly: ASM1815329v1), *D. rubida* (assembly: ASM3504616v1), *M. domestica* (assembly: Musca_domestica.polishedcontigs.V.1.1), and *L. cuprina* (assembly: ASM2204524v1) (Supplementary File 1 Table A).

### Ensembl ortholog analysis

We used the Ensembl Compara online ortholog tool (Kersey et al. 2010 and Accessed date: June 2022) to collect ortholog predictions for 366 GSC-regulating genes for the 10 *Drosophila* species that were available in Ensemble Compara (*D. melanogaster*, *D. simulans*, *D. sechellia*, *D. yakuba*, *D. ananassae*, *D. pseudoobscura*, *D. willistoni*, *D. mojavensis*, *D. virilis*, *D. grimshawi*) and two outgroup species (*L. cuprina* and *M. domestica*) (Supplementary File 2 Tables A and B). This tool catalogs relevant information about each ortholog including sequence alignment, target percent ID (percentage of orthologous sequence matching the *D. melanogaster* sequence), query percent ID (percentage of *D. melanogaster* sequence matching the orthologous sequence), gene order conservation score (evaluating synteny), and high or low ortholog confidence (calculated using results from other categories) (Kersey et al. 2010). Predicted orthologs were included as confident predictions if sequence alignment is equal to or greater than 25% identity (github.com/lukearnce/bam_null_ortholog).

### RBBH ortholog pipeline

We implemented the following multistage pipeline to execute our RBBH ortholog analysis.

1 Initial RBBH

We first downloaded highly contiguous, well-annotated long-read sequences from NCBI for all included species (15 *Drosophila* species plus the two outgroup species) (Kim et al. 2021; Li et al. 2021). *D. teissieri*, *D. takahashii*, *D. elegans*, *D. serrata*, *D. obscura*, *D. rubida*, *M. domestica*, and *L. cuprina* were added to the initial set of species from Ensembl Compara. Then, we used custom scripts to perform forward and reverse BLASTp searches to identify potential orthologs and filter ortholog hits for GSC-regulating genes as well as GSC gene interaction network genes (Supplementary File 2, github.com/lukearnce/bam_null_ortholog).

2 RBBH + syntenic evaluation

For genes with predicted absences, we conducted forward and reverse BLASTn searches as well as reciprocal BLASTp searches for the gene predicted absent and the syntenic genes (three on each side) flanking the GSC gene in *D. melanogaster*. We executed this to search for genes that are actually present but may have been predicted absent due to location at the end of contigs (Supplementary Files 2 and 3, github.com/lukearnce/bam_null_ortholog).

3 Direct validation of predicted gene ortholog absences

We identified 21 predicted GSC gene ortholog absences with retained syntenic blocks (at least one syntenic gene remaining from each side of the ortholog absence) and evaluated 17 of them directly via sequencing (Supplementary File 1). The other four had prohibitively large gaps. We developed primers (IDT) anchored in the CDS of syntenic genes for PCR amplification of the sequence between retained syntenic genes. We then gel extracted, purified, and sequenced PCR products to directly verify gene absence (Supplementary Files 1–3, github.com/lukearnce/bam_null_ortholog).

### Interaction networks and functional information

We cataloged physical interactors and genetic interactors from Flybase datasets for GSC-regulating genes with predicted absences and evaluated interaction network genes for orthologs across included species using the same RBBH pipeline (Choi and Aquadro 2015) as well as ortholog predictions from Li et al. (2021). Annotated molecular functions (Choi and Aquadro 2015), functional categories identified by complex-enrichment analysis of the 366 GSC genes, and defect type, defined by the observed phenotypic effect of RNAi knockdown, were also incorporated from Yan et al. (2014). Defect types include GSC loss (cell viability), GSC loss (agametic), differentiation defect, and oocyte-specific phenotypes/late oogenesis (Supplementary File 3).

## Results

### No evidence for positive selection due to amino acid diversification at *bam* in *D. americana*

**Fig. 2.**
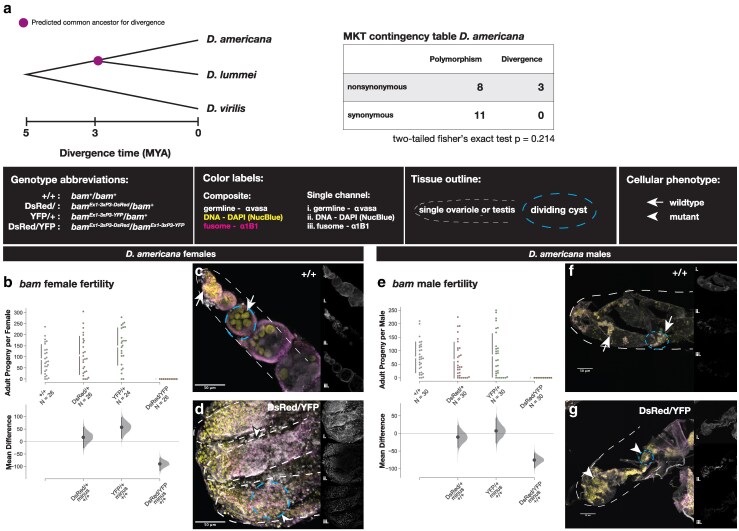
Fertility and cytological analyses of *bam* function in adult *D. americana.* Fertility is presented as adult *D. americana* progeny per fly for each *bam* genotype and presented separately for females (b) and males (e). The raw data as progeny per fly are plotted on the upper axes with the mean difference for the three genotype comparisons against the shared control wild type illustrated in the Cumming estimation plots on the lower axes. Mean differences are plotted as bootstrap resampling distributions. Each mean difference is depicted as a dot, and 95% confidence intervals are indicated by the vertical black bars. Immunostaining of ovaries (b and c) and testes (e and f) of wild type (b and e) and null *bam* genotypes (c and f). Composite Z-projections for ovaries and testes show staining for the germline (vasa), fusome (1B1), and nuclei (DAPI) with separate single channels for each image illustrated in the side panels (i. vasa, ii. DAPI, iii. 1B1). Wild-type tissue phenotypes are indicated with arrows and mutant tissue phenotypes are indicated with arrowheads. Dashed cyan ovals indicate dividing tumorous (null) and nontumorous (wild-type) cysts.

In the species for which *bam* function has been analyzed, species with evidence of positive selection also require *bam* for oogenesis (*D. melanogaster*, *D. simulans*, *D. yakuba*, *D. ananassae*). However, in *D. teissieri*, there is no evidence of positive selection at *bam* and *bam* is not required for oogenesis. To further define the evolutionary history of *bam's* function in gametogenesis, we first assessed patterns of polymorphism and lineage-specific divergence at *bam* in *D. americana*, a representative of a lineage divergent from *D. melanogaster*, using the MKT (Fig. 2a). We failed to reject the null hypothesis of neutrality, suggesting *bam* is not likely evolving under positive selection for amino acid diversification in *D. americana.*

### *Bam* is necessary for female and male fertility and germ cell differentiation in *D. americana*

Next, we used CRISPR/Cas9 gene editing to generate *bam* null alleles in *D. americana*. Wild-type (+/+) and *bam* null heterozygotes (*bam*^3xP3−Dsred^/*bam^+^* and *bam*^3xP3−YFP^/*bam^+^*) are fertile in both males and females (Fig. 2b and e). However, *bam* null homozygotes (*bam*^3xP3−Dsred^/*bam^3xP3−YFP^*) were completely sterile in males and females (*P* < 0.0001, permutation test, Fig. 2b and e) (github.com/lukearnce/bam_null_ortholog). One copy of wild-type *bam* is sufficient to rescue the *bam* null sterility phenotype in *D. americana*, as is the case for both females and males in *D. melanogaster*, *D. simulans*, and *D. yakuba* and for females in *D. ananassae* (Bubnell, et al. 2022).

To confirm the *bam* null sterile fertility phenotype was due to defects in GSC function as expected if *bam* function is conserved between *D. melanogaster* and *D. americana*, we evaluated the cytology of *D. americana bam* null ovaries and testes (Fig. 2). We imaged 3–5-d-old ovaries from *D. americana bam* wild-type and *bam* null females that we immunostained with antibodies to vasa and 1B1 and mounted with DAPI. Homozygous *bam* null cytology recapitulated the classic *bag of marbles* phenotype, with overproliferation of small, undifferentiated GSC-like cells in the ovaries (Fig. 2d) and testes (Fig. 2g) in contrast to *bam* wild-type ovaries (Fig. 2c) and testes (Fig. 2f) which consist of cysts made of larger differentiating germline cells. The cysts consisting of tumorous (null genotype) and nontumorous (wild-type genotype) are outlined in cyan. *bam* null ovaries do not contain any cysts with differentiating nurse cells as outlined for wild-type *bam* ovaries. Additionally, the tissue is much smaller as it lacks any differentiating germline cells (Fig. 2c and d, scale bar). Cysts in *bam* null testes consist of many small, undifferentiated cells, with the tissue also smaller in size compared to the wild-type tissue which consists of cysts made up of differentiating spermatogonial cells (Fig. 2f and g, cyan outline, scale bar). Our cytological data reveal that *bam* is necessary for early germ cell differentiation in *D. americana*, consistent with the fertility assay results (Fig. 2a and d).

### Essential GSC gene ortholog absences across species

While functional genetic analyses for *bam* across diverse lineages in the genus *Drosophila* revealed some striking variation in *bam's* role in fertility and germ cell differentiation, the financial and time costs required to generate null mutants for other GSC genes across diverse species are prohibitive at this point. Therefore, we next chose an alternative, albeit less sensitive, approach to evaluate the functional consistency of the roles of GSC-regulating genes that are essential in *D. melanogaster*. Focusing on the experimentally defined set of GSC-regulating genes determined to be essential in *D. melanogaster* (Yan et al. 2014), we defined a functional difference in GSC regulation pathways between species if an essential ortholog in *D. melanogaster* was absent other *Drosophila* and/or outgroup species.

**Fig. 3.**
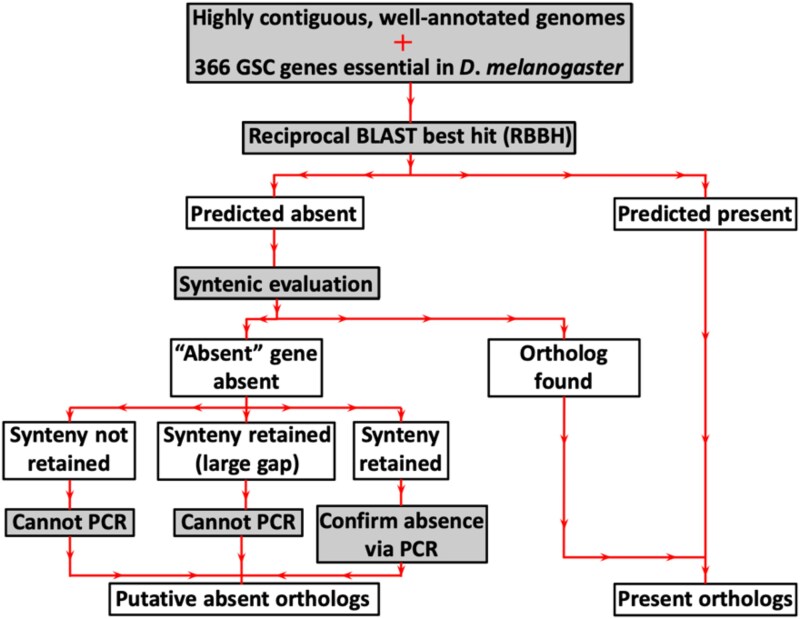
Flowchart of the ortholog search pipeline including syntenic evaluation.

**Fig. 4.**
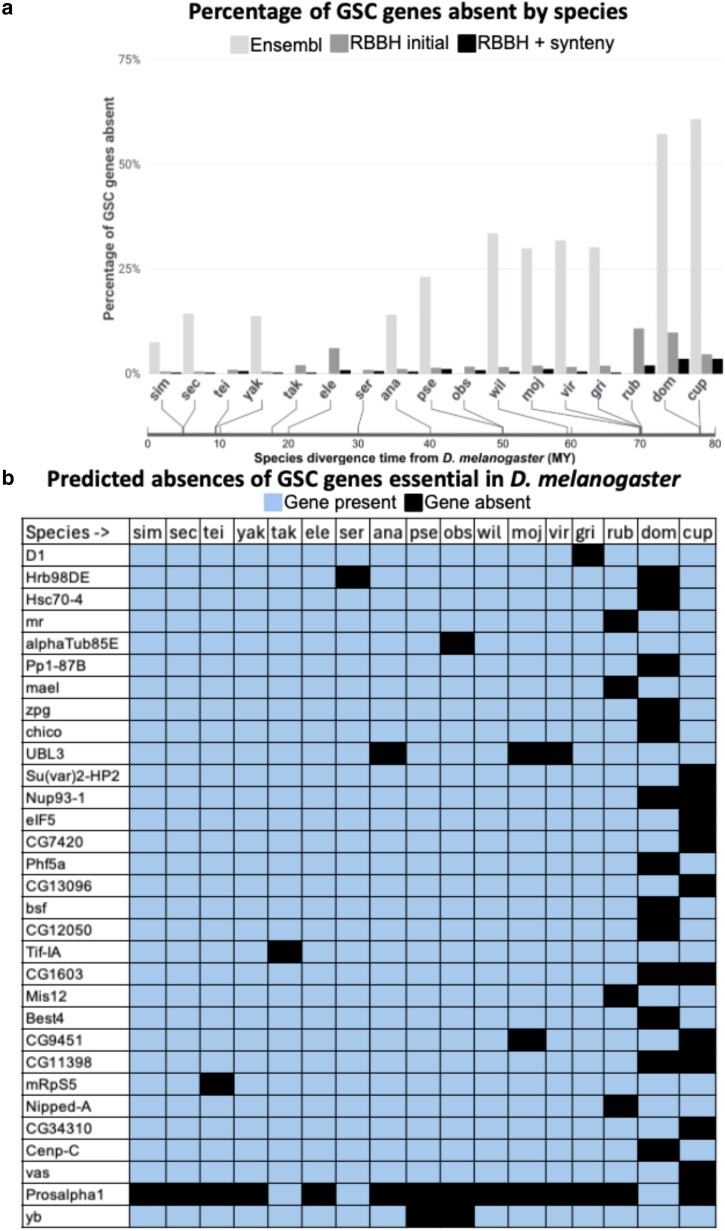
Predicted presence/absences in 15 increasingly divergent *Drosophila* species and two outgroups (*M. domestica* and *L. cuprina*) of 366 GSC-regulating genes essential in *D. melanogaster* (predicted by Yan et al. 2014) by species plus *bgcn* and *Yb* using our multiple ortholog detection strategies shown in Fig. 3. a) The percentage of GSC genes predicted absent by species for three ortholog identification strategies. Light gray bars represent ortholog predictions using Ensembl, dark gray represent predictions with RBBH alone, and black represent RBBH plus syntenic evaluation. The bar below the species indicates divergence time from *D. melanogaster* as detailed in Fig. 1. b) The presence and absence of GSC genes across 15 *Drosophila* and two outgroup species that are predicted absent in at least one species after RBBH and syntenic evaluation. Blue indicates gene presence and black indicates gene absence.

Our initial Ensembl ortholog analysis predicted absences for 311 of 366 GSC-regulating genes for nine *Drosophila* and two outgroup species (Supplementary File 2 Supplementary Tables A and B, github.com/lukearnce/bam_null_ortholog). The number of absences per species ranged from 27 (7.38%) in *D. simulans* to 222 (60.66%) in the outgroup species *L. cuprina* (sheep blowfly). Next, we used our RBBH ortholog assessment (Fig. 3) that revealed predicted absences for 79 of 366 GSC-regulating genes across 15 *Drosophila* and two outgroup species. The number of absences per species ranged from 2 (0.5%) in *D. simulans* to 39 (10.7%) in *D. rubida*. Finally, with our most stringent approach combining RBBH and syntenic evaluation of orthologs, we predicted absences for 30 of 366 GSC-regulating genes across 15 *Drosophila* and two outgroup species (Fig. 4) (Supplementary File 3, github.com/lukearnce/bam_null_ortholog). Our most of stringently assessed number absences per species ranged from 1 (0.27%) in *D. simulans* to 13 (3.6%) in the outgroup species *M. domestica* (house fly).

### Verification of gene absences with retained syntenic blocks

To experimentally confirm the predicted GSC-regulating gene ortholog absences we found with RBBH + syntenic evaluation with retained syntenic blocks, we PCR amplified and sequenced the syntenic block spanning the expected gene absence. The PCR sequencing results verified the syntenic flanking sequences are present and the gene itself between them is absent for all predicted absences with retained synteny therefore our RBBH + syntenic computational predictions represented real absences in the amplified regions (github.com/lukearnce/bam_null_ortholog). Using our final ortholog presence and absence results, we then sought to identify potential patterns in gene presence or absence across the included species. Primarily, we evaluated whether and in what ways essential gene conservation varied across gene functional categories and interaction network size (Supplementary File 3).

### Variable GSC gene conservation across species and functional categories

**Fig. 5.**
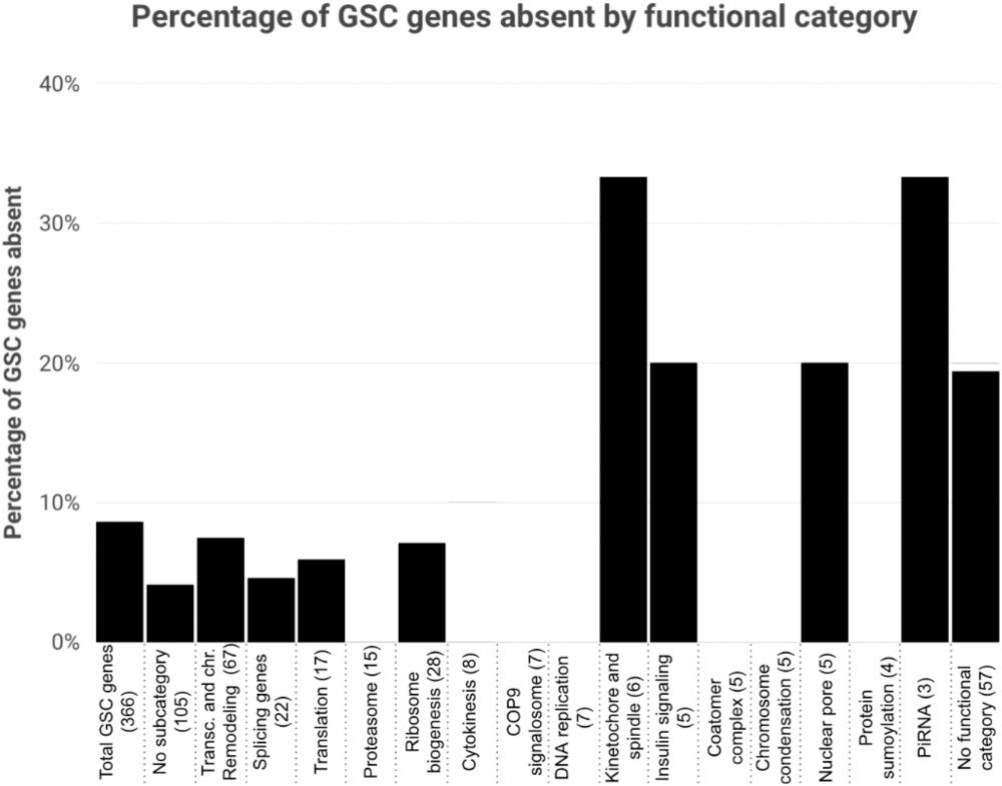
The percentage of GSC genes absent by functional category. Categories are pulled from the gene interaction network generated in Yan et al. (2014). The “No subcategory (105)” group includes genes represented in the interaction network map without clear categorical associations and the “No functional category (57)” includes GSC genes that do not appear in the interaction network map.

We found that GSC-regulating genes with predicted absences are unequally represented across functional categories identified by complex enrichment. At the extremes, there are zero predicted absences in the proteasome functional category (15 genes) and two of six genes (33%) in the Kinetochore and spindle functional category (Fig. 5). Absences categorized by defect types show less variation ranging from 7.14% for GSC loss (cell viability) (12 of 168) to 11.11% for genes with oocyte-specific phenotypes/late oogenesis (5 of 45).

### Variability in absent GSC gene interaction network size and absences

**Fig. 6.**
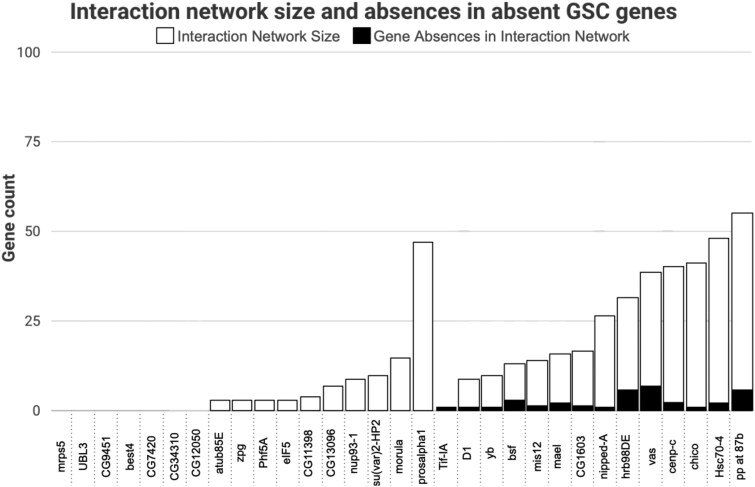
Interaction network size and absences in absent GSC genes. Genes with at least one absence in the included species are listed with white bars representing the size of their interaction networks including genetic and physical interactors. The number of interaction network genes absent are represented with black bars.

**Fig. 7.**
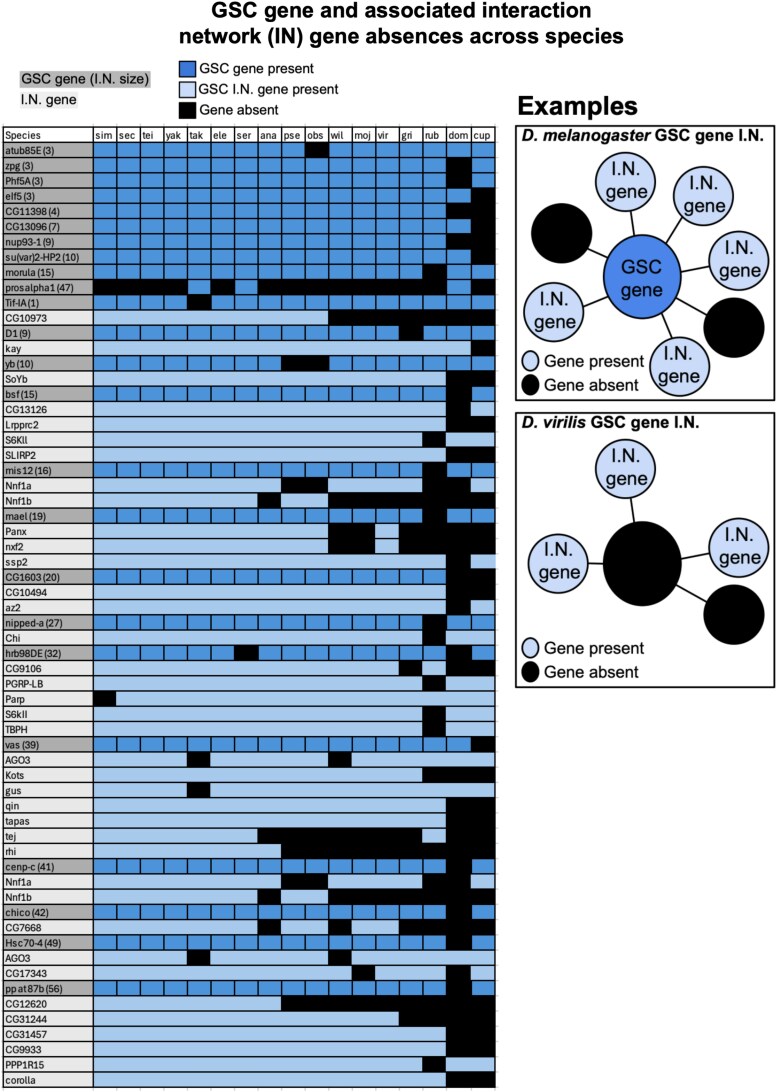
GSC gene and associated network absences across species. GSC genes with absences in at least one included species are highlighted in dark gray. Associated interaction network (I.N.) genes are indented, italicized, and highlighted in light gray. Gene presence is indicated by light blue and absence is indicated by black. First, GSC genes with no absences in their interaction networks are arranged by increasing interaction network size. Next, GSC genes with absences in their interaction networks are arranged in the same manner. GSC genes with no interaction networks (six genes) are excluded from this figure.

Of the 30 GSC-regulating genes (from the 366 in Yan et al. [2014]) with predicted absences across included species, 23 genes have physical and/or genetic interactions (Fig. 6, Supplementary File 3). Thirteen of these 23 genes also have predicted absences in their interaction networks, and most of these interaction network absences are in the same species as their related absent GSC-regulating gene (Fig. 7, Supplementary File 3).

## Discussion

Fertility and germline cytological assays of *bam* null mutants in *D. americana* reveal that *bam* is essential for GSC differentiation in both males and females. This demonstrates that *bam*'s essential function in GSC differentiation is not novel to the *D. melanogaster* species group, and its evolutionary origin in this function likely occurred just prior to or just after the origin of the genus *Drosophila*. *Bam* functional differences in *D. teissieri* and *D. ananassae* likely represent lineage-specific GSC functional losses. Similar losses may exist among the many other nontested taxa in this species-rich genus. These analyses of *bam* function show that high amino acid sequence variability between species does not necessarily imply functional divergence. *D. americana bam* and *D. melanogaster bam* share only 35% sequence identity, yet both execute the same essential function in GSC daughter differentiation. In contrast, *D. teissieri bam* and *D. melanogaster bam* share 75% sequence identity and they are functionally distinct.

Conservation of sequence does not necessarily imply conservation of function and divergence of sequence does not necessarily imply a divergence in function. A classic example of this dynamic includes cytochrome c (Garrido et al. 2006). Cytochrome c is one of the most conserved proteins among eukaryotes with an amino acid sequence conservation of 70% to 90% between species as divergent as yeast and mammals. In many yeasts and lower eukaryotes, cytochrome c functions solely as an electron carrier in the mitochondrial respiratory chain while in mammals the same (or nearly identical) cytochrome c also acquired an additional role as an apoptotic signal by helping to trigger caspase activation and programmed cell death. This extra “moonlighting” function in apoptosis is a dramatic divergence in biological role despite a highly conserved structure (Garrido et al. 2006).

The resolution and depth of our analysis was made possible by to the large collection and phylogenetic density of high-quality genomes for a growing number of species in the genus *Drosophila* in combination with the depth of functionally defined genes within *D. melanogaster*. Large-scale gene ortholog identification is mostly not possible in species with only low-quality genomes available, and, potentially as a result, there are currently very few large-scale comparative analyses of gene orthologs, essential or nonessential (Bergmiller et al. 2012; Carranza et al. 2018; Leeuwen et al. 2020; Deng et al. 2024). However, as more high-quality genomes are produced and published for more species, this type of comparative ortholog analysis becomes more feasible.

Even when using only species with available high-quality genomes for comparative analysis, efforts must be made to ensure potential orthologs and absences are properly identified. For all the GSC-regulating genes in *D. melanogaster* that we analyzed (366 from Yan et al. [2014]) plus *bgcn* and *Yb*, ortholog predictions vary dramatically based on identification strategy. Using Ensembl alone, we predicted 85% (311 of 366) of GSC-regulating genes absent in at least one included species while we only predicted ∼8% (30 of 366) absent based on the more detailed evaluation incorporating RBBH and synteny. Due to the significant discrepancy in predictions, the predicted presence or absence of orthologs via Ensembl must be further interrogated by additional strategies including RBBH and syntenic evaluation before confidently characterizing predictions as orthologs that are truly absent. As an example, benign gonial cell neoplasm (*bgcn*) is a crucial gene that is broadly conserved, including a human ortholog, that was predicted to be absent in the initial RBBH evaluation. However, using follow-up BLASTn and syntenic evaluation, we found the *bgcn* ortholog split over short contigs that made initial identification difficult. In contrast, *Yb*, an additional essential GSC-regulating gene in *D. melanogaster*, was computationally predicted by our pipeline to be absent in *D. obscura* and *D. pseudoobscura*. Follow-up syntenic evaluation and sequencing confirmed these absences of *Yb* from their syntenic regions in these two species (the results for *D. pseudoobscura* being consistent with those from Chary and Hayash [2023]).

Final ortholog absence predictions were further evaluated for confidence by consideration of several characteristics: direct validation of the absence, divergence time of the species from *D. melanogaster*, and the presence of gene orthologs in more or equally divergent species. We have the highest confidence in predicted ortholog absences for genes that were verified absent via PCR sequencing (17 genes [Supplementary File 1, github]). Due to the significant divergence time between *D. melanogaster* and outgroup species *M. domestica* and *L. cuprina*, we consider predicted absences in these species to be lower confidence given the higher possibility of significant sequence divergence leading to ortholog detection failure.

Failure to detect orthologs that are present, but highly divergent in sequence, is a legitimate concern when executing comparative analysis of gene orthologs across several significantly diverged species (Weisman 2020). One metric that can provide more support for ortholog absence rather than detection failure is the presence of an ortholog of the same gene in a more divergent lineage. Of the 30 GSC genes with predicted absences, all but three have present orthologs in more or equally divergent lineages and absences within closer lineages (Nup93-1, CG1603, CG11398) which provides supporting evidence for gene loss rather than detection failure. Because of this, we consider the 27 to be of highest confidence. This is not the case for interaction network genes. Of the 38 interaction network genes with predicted absences, 17 have present orthologs in more or equally divergent lineages and absences within closer lineages. We have less of an ability to rule out detection failure for GSC interaction network genes, but, to the extent that our estimates are accurate, there is a tendency toward interaction network absences. There can be large interaction networks with no absences, but the data suggest the genes that are absent tend to occur in larger interaction networks. As more genomes become available, this can be tested more rigorously.

The 30 (out of 366) GSC-regulating genes that are predicted to be absent by our pipeline represent significant flexibility in essential GSC-regulating genes beyond *bam* across species. Consideration of only the highest confidence predictions leads to qualitatively similar conclusions. These results add to a growing body of evidence that suggests the general ortholog assumption of shared function is not universal and provide examples of DSD in a process critical for early reproduction.

These 30 genes are also not equally distributed across functional categories. While there are no predicted absences in proteasome genes, two of six (33%) genes involved in the kinetochore and spindle have predicted absences. This variable flexibility across functional categories suggests there may be some networks that are more flexible in their regulation, while others are particularly intolerant. Additionally, further analysis of these 30 genes with absent orthologs shows that these genes generally have interaction networks including genetic and physical interactors. Absences in orthologs of interaction network genes also generally occur in the same species that their related GSC gene is absent. This could potentially indicate that having a network of interacting partners more easily enables gene loss by compensating for the function of the absent gene through redundancy or alternative regulation, while essential genes without large interaction networks could generally be more difficult to replace (Carroll 2008; Wagner 2008; Albalat and Canestro 2016). One gene, *Prosalpha1*, is absent in the most species of the included GSC genes. In addition to having a large interaction network (47 genes), it also has a very similar gene, *Prosalpha1R*, within the surrounding syntenic region. *Prosalpha1R* could possibly facilitate alternative execution of *Prosalpha1*'s essential function.

Our findings here provide additional evidence that orthologous genes do not necessarily function identically even when critical for regulating essential systems. Despite the essential nature of proper gamete development, what has been described as DSD is occurring in genes critical for regulating the earliest stages of this process across *Drosophila* and closely related outgroups. Spermatogenesis regulating genes further downstream of GSC regulation have also been evaluated across some *Drosophila* species. Results showcase similar functional flexibility with duplications or losses of sperm nuclear basic proteins as well as the emergence of many functional de novo genes (Chang et al. 2023; Lee U 2025). Some of the particular genes involved in the process change, but the final phenotype is maintained across species.

We conclude that ortholog function must be evaluated in a species-specific manner. These 30 GSC genes and *Yb* that show absences in some species represent the baseline of functional flexibility for the *D. melanogaster*-essential GSC-regulating genes, but many other GSC-regulating genes still present, like *bam*, could have variable function across species. The extent of DSD occurring beyond GSC-regulating genes in these 17 species is largely unknown, but further evaluation of ortholog functional flexibility focusing on different organisms and phenotypes could reveal differences in the key genes regulating any number of traits across species. Results from this dataset suggest some potential patterns related to ortholog loss of essential genes, but more extensive analysis needs to be executed to make more concrete assessments of the prevalence and characteristics of ortholog functional flexibility.

## Supplementary Material

jkaf224_Supplementary_Data

## Data Availability

All raw and filtered data are available in a public repository at www.github.com/lukearnce/bam_null_ortholog, archived at https://doi.org/10.5281/zenodo.17123061. These data include the following: MKT for *D. americana bam* PRANK inputs and control parameters for the *D. americana bam* MK test with contingency table values and the *P*-value of the Fisher's exact test. *D. americana bam* CRISPR Null fertility assays *D. americana* male and female estimation statistics and significance as an effect size outside of the 95% confidence intervals GSC ortholog identification Initial RBBH ortholog analysis results, full genomes, custom scripts to execute forward and reverse BLASTp, filtered ortholog hits, interaction network genes, RBBH with syntenic evaluation gene hits, and sequence verification of predicted ortholog absences Supplemental material available at G3 online.
